# Deficiency of tPA Exacerbates White Matter Damage, Neuroinflammation, Glymphatic Dysfunction and Cognitive Dysfunction in Aging Mice

**DOI:** 10.14336/AD.2018.0816

**Published:** 2019-08-01

**Authors:** Peng Yu, Poornima Venkat, Michael Chopp, Alex Zacharek, Yi Shen, Linlin Liang, Julie Landschoot-Ward, Zhongwu Liu, Rongcai Jiang, Jieli Chen

**Affiliations:** ^1^Department of Neurosurgery, Tianjin Medical University General Hospital, and Tianjin Neurological institute, Key Laboratory of Post-Neurotrauma Neurorepair and Regeneration in Central Nervous System, Ministry of Education and Tianjin City, Tianjin, China.; ^2^Neurology, Henry Ford Hospital, Detroit, MI, USA.; ^3^Department of Neurosurgery, Shanghai Tenth People’s Hospital, Tongji University, Shanghai, China.; ^4^Department of Physics, Oakland University, Rochester, MI, USA.; ^5^Reproductive Medical Center, Henan Provincial People’s Hospital, Zhengzhou, China

**Keywords:** blood brain barrier, cognitive function, glymphatic system, thrombosis, tPA, white matter

## Abstract

Tissue plasminogen activator (tPA) is a serine protease primarily involved in mediating thrombus breakdown and regulating catabolism of amyloid-beta (Aβ). The aim of this study is to investigate age-dependent decline of endogenous tPA and the effects of tPA decline on glymphatic function and cognitive outcome in mice. Male, young (3m), adult (6m) and middle-aged (12m) C57/BL6 (wild type) and tPA knockout (tPA^-/-^) mice were subject to a battery of cognitive tests and white matter (WM) integrity, neuroinflammation, and glymphatic function were evaluated. Adult WT mice exhibit significantly decreased brain tPA level compared to young WT mice and middle-aged WT mice have significantly lower brain tPA levels than young and adult WT mice. Middle-aged WT mice exhibit significant neuroinflammation, reduced WM integrity and increased thrombin deposition compared to young and adult mice, and increased blood brain barrier (BBB) permeability and reduced cognitive ability compared to young WT mice. In comparison to adult WT mice, adult tPA^-/-^ mice exhibit significant BBB leakage, decreased dendritic spine density, increased thrombin deposition, neuroinflammation, and impaired functioning of the glymphatic system. Compared to age-matched WT mice, adult and middle-aged tPA^-/-^ mice exhibit significantly increased D-Dimer expression and decreased perivascular Aquaporin-4 expression. Compared to age-matched WT mice, young, adult and middle-aged tPA^-/-^ mice exhibit significant cognitive impairment, axonal damage, and increased deposition of amyloid precursor protein (APP), Aβ, and fibrin. Endogenous tPA may play an important role in contributing to aging induced cognitive decline, axonal/WM damage, BBB disruption and glymphatic dysfunction in the brain.

Tissue plasminogen activator (tPA) is a serine protease primarily involved in mediating thrombus breakdown. As a therapeutic agent, tPA is an effective and approved Food and Drug Administration (FDA) treatment for acute ischemic stroke. tPA converts inactive plasminogen to the active protease plasmin which dissolves fibrin clots into soluble degradation products [1]. However, growing evidence implies that tPA has a close relationship with cognitive function and dementia [2]. tPA also initiates the degradation of amyloid-β (Aβ), resulting in decreasing level of Aβ derived from amyloid precursor protein (APP), which may delay pathogenic progression of Alzheimer's disease (AD). With advancing age, tPA proteolytic activity is reduced and Aβ deposition increases [2].

Endogenous tPA plays an important role in maintaining both high fibrinolytic activity and effective clot lysis on the vascular endothelial cell surface [3]. Fibrinolysis prevents unnecessary accumulation of intravascular fibrin and enables the removal of thrombin [4]. Thrombin, known as a serine protease, has been shown to have a close relationship with cognitive defects after traumatic brain injury [5]. Fibrin, transformed from fibrinogen, is an essential plasma protein for clot formation. Increased fibrin deposition and clotting that is resistant to fibrinolysis are found in AD brain parenchyma and vessels [6]. Fibrin co-localizes and interacts with Aβ and reduces degradation of Aβ [6, 7]. Neuronal and synaptic degeneration and chronic inflammation are often present in the vicinity of fibrin deposition which may contribute to the pathophysiology of dementia [8]. AD patients also suffer from decreased cerebral blood flow due to blocking of cerebral blood vessels and capillaries by fibrin deposits [9]. In middle-aged rats, significant cognitive impairment and increased density of fibrin/fibrinogen immunoreactive vessels in the ischemic brain are observed in diabetes and diabetic stroke rats compared to non-diabetic control and non-diabetic-stroke rats, respectively [10]. In a multi-city cohort comprising elderly men and women >65 years of age, elevated fibrinogen and D-Dimer levels were associated with incident arterial disease and identified as a risk factor for vascular dementia [11].

The glymphatic system is a waste clearance pathway in the brain where interaction between the cerebrospinal fluid and interstitial fluid enables clearance of metabolic wastes, toxins and other solutes from the brain parenchyma [12]. Glymphatic dysfunction has been demonstrated in aging rodent brain [13] and could potentially lead to Aβ accumulation, a hallmark of AD pathology [14]. Impaired glymphatic clearance in the aging brain may lead to neurodegeneration characterized by misaggregation of detrimental proteins [15]. Water channel aquaporin-4 (AQP4), which is expressed predominantly on perivascular astrocytic end feet, plays an essential role in functioning of the glymphatic system [16]. Previous studies have shown that microinfarction in the rodent brain was associated with cognitive decline, WM damage and glymphatic dysfunction [17, 18].

In this study, we hypothesize that aging leads to decreased endogenous cerebral tissue tPA levels and that tPA plays an important role in cognitive function. Deficiency of brain tPA with age may increase fibrin and Aβ deposition in the brain, as well as induce glymphatic system dysfunction which in concert lead to cognitive deficits.

## MATERIALS AND METHODS

All experiments were conducted in accordance with the standards and procedures of the American Council on Animal Care and Institutional Animal Care and Use Committee of Henry Ford Health System.

### Experimental groups

tPA knockdown mice (tPA^-/-^) were purchased from Jackson Laboratories (Bar Harbor, ME) and the tPA^-/-^ mouse colony was expanded following homozygous mating. Since the tPA^-/-^ mouse line has C57BL/6J background, we purchased age-matched C57BL/6J mice from Jackson Laboratories (Bar Harbor, ME) as wild type control (WT). Male WT and tPA^-/-^ mice of varying age- 3 months (young), 6 months (adult), and 12 months (middle-aged) (n=6/group), were subject to a battery of cognitive tests and then euthanized for immunostaining quantification analysis and PCR and ELISA studies [19]. Additional sets of adult mice were prepared for Golgi staining (n=6/group) and glymphatic system measurement (n=18/group).

### Cognitive function tests

A battery of cognitive tests was performed by an investigator who was blinded to the experimental groups using previously described methods [17]. Novel object recognition test was used for evaluating short term visual memory and learning [20]. Odor test was employed to evaluate olfactory learning and memory [21]. Morris water maze (MWM) test was performed to test visual and spatial learning and memory [22].

### Immunohistochemical analysis

Mice were sacrificed and transcardially perfused with 0.9% saline and brains were immediately removed and fixed in 4% paraformaldehyde. Coronal brain tissue sections were prepared for immunostaining. Antibody against FITC (Fluorescein isothiocyanate) labeled AQP4 (Aquaporin-4, rbt, ab3594, EMD Millipore, Billerica, MA, 1:1500), APP (rbt, 2452, Cell Signaling, Danvers, MA, 1:50), IBA1 (rbt, 019-19741, Wako, Mountain View, CA, 1:1000), Fibrin b (mo, 350, Sekisui Diagnostics, Lexington, MA, 1:35), Von Willebrand Factor (vWF, rbt, A0082, Dako, Santa Clara, CA, 1:400), Glial fibrillary acidic protein (GFAP, rbt, Z0334, Dako, Santa Clare, CA, 1:10,000), Albumin-FITC (ab53435, Abcam, Cambridge, MA, 1:500), Thrombin (mo, sc-13503, Santa Cruz, Dallas, TX, 1:500), CD68 "ED1" (mo, MCA 341R, ABD Serotec, Raleigh, NC, 1:30) and Cy3 labeled antibody against Aβ (Amyloid beta 1-42, rbt, ab10148, Abcam, Cambridge, MA, 1:100) were used. Bielschowsky-silver (BS, axon marker), luxol fast blue (LFB, myelin marker) staining were employed to evaluate axon and myelin density, respectively [23]. DAPI counter stain was used to stain nuclei. Control experiments consisted of staining brain coronal tissue sections as outlined above, but non-immune serum was substituted for the primary antibody.

**Figure 1.**
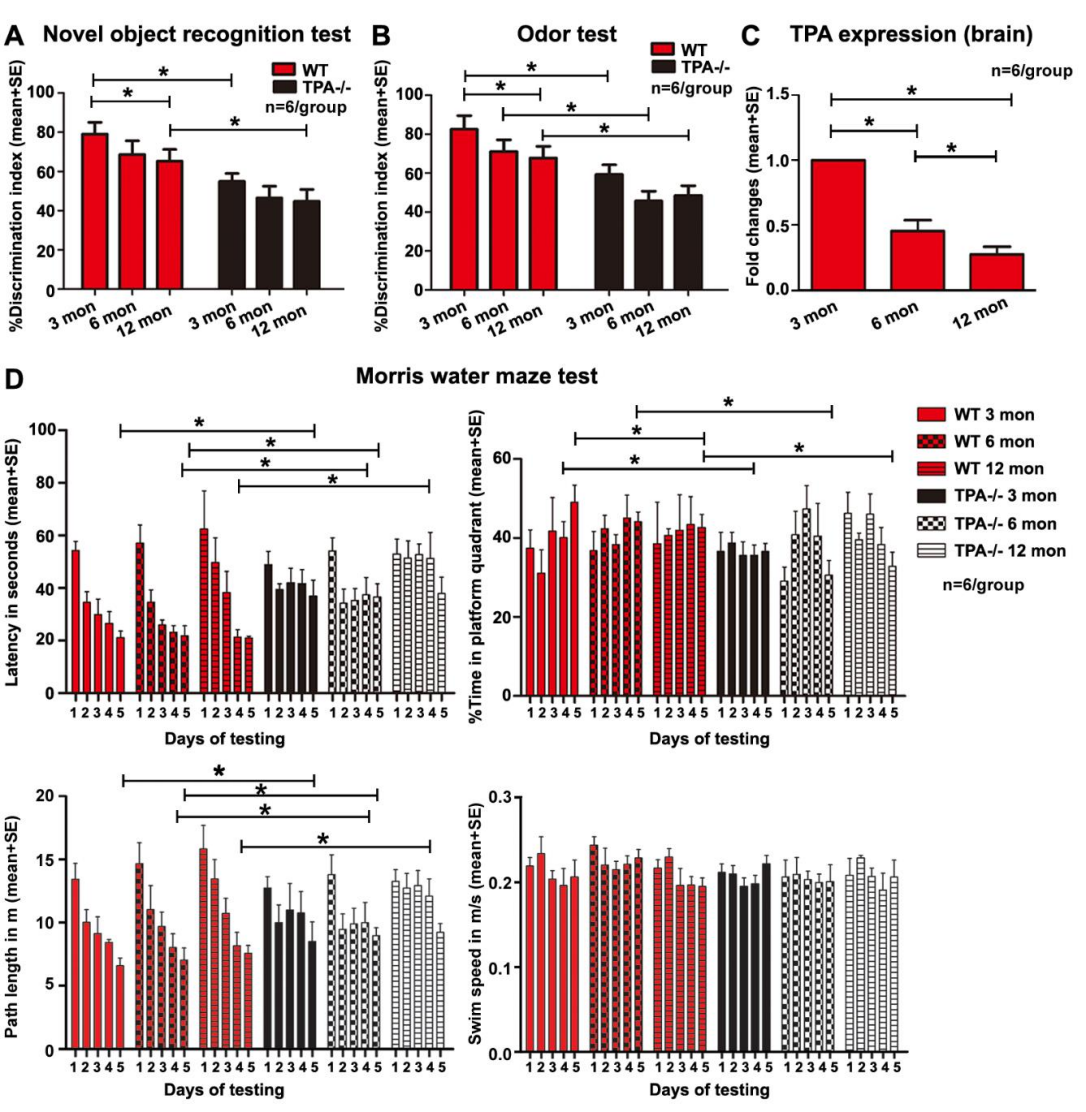
Aging and tPA deficiency induce progressive cognitive deficits (**A**) Significant short-term memory loss evaluated by novel object recognition test is evident in middle-aged WT mice compared to young WT mice, and in young and middle-aged tPA^-/-^ mice compared to age-matched WT mice. (**B**) Long-term memory deficits evaluated by odor test are evident in middle-aged WT mice compared to young WT mice as well as young, adult and middle-aged tPA^-/-^ mice compared to corresponding age-matched WT mice. (**C**) Aging decreases tPA expression level in brain of WT mice. Adult mice (6m) have significantly lower tPA expression in brain compared to young mice (3m), and middle-aged (12m) mice have significantly lower tPA levels than young and adult mice. (**D**) tPA^-/-^ mice and middle-aged WT mice exhibit significantly increased spatial learning and memory deficits measured by Morris Water maze test. *p<0.05, n=6/group.

**Figure 2.**
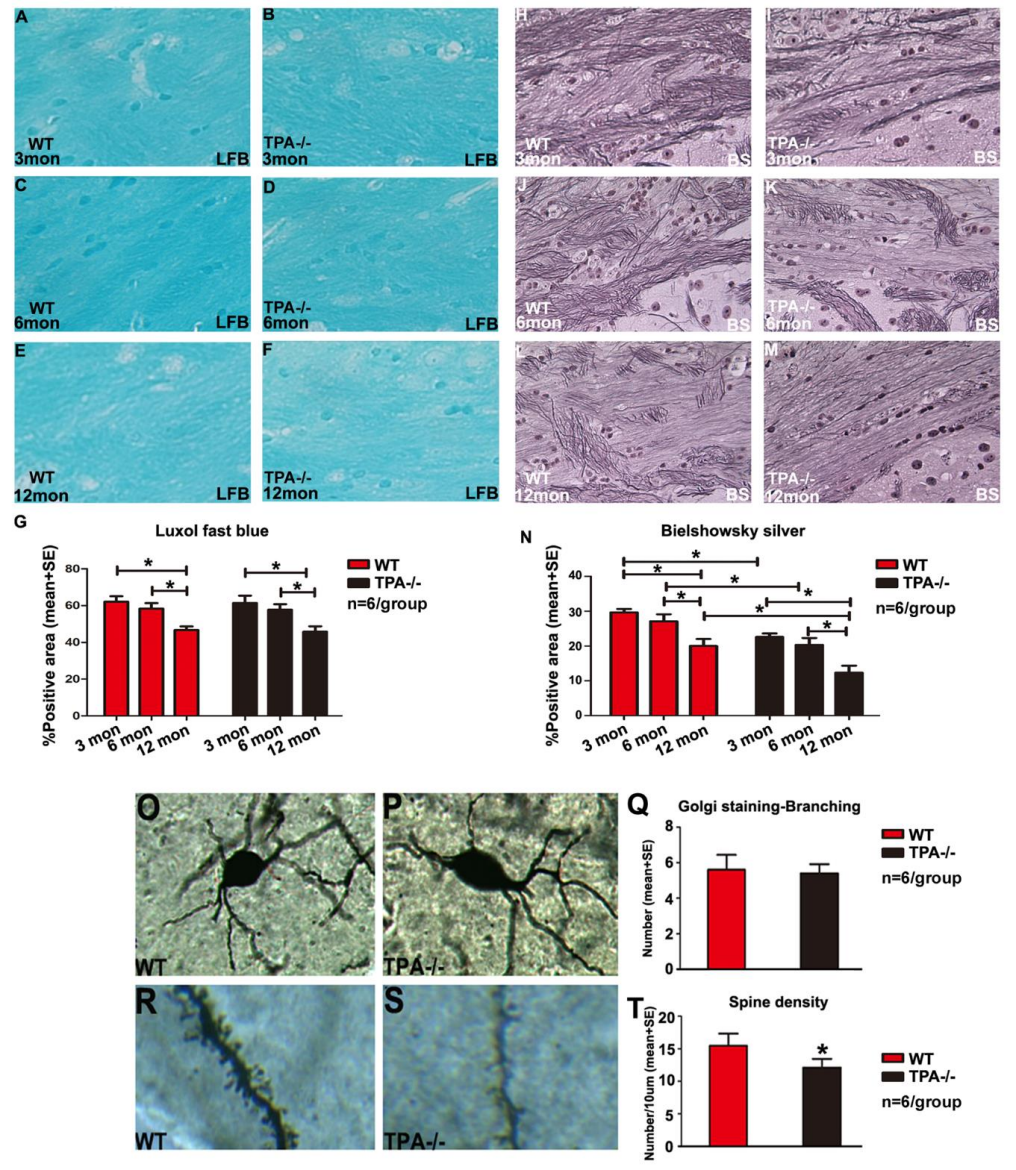
Aging and deficiency of tPA induce WM damage and loss of synaptic plasticity Middle-aged WT and tPA^-/-^ mice exhibit significantly increased myelin rarefaction as indicated by Luxol fast blue immunostaining (**A-F**) compared to young WT and young tPA^-/-^ mice, respectively. Luxol fast blue quantification data is presented in panel (**G**. **H-M**) Middle-aged WT and tPA^-/-^ mice exhibit significantly increased axon damage indicated by decreased axon density in Bielschowsky silver immunostaining compared to young WT and young tPA^-/-^ mice respectively. Young, adult and middle-aged tPA^-/-^ mice exhibit significantly increased axonal damage compared with age-matched WT mice. Bielschowsky silver quantification data are presented in panel N. (**O-Q**) Analysis of Golgi staining under a 40× objective indicates that there were no changes in the primary neuronal branching in tPA^-/-^ mice compared to WT mice. (**R-T**) Analysis of Golgi staining under a 100× objective indicates that the spine density in cortical neurons was significantly decreased in adult tPA^-/-^ mice compared to adult WT mice. *p<0.05, n=6/group.

### Quantification analysis

An investigator blinded to the experimental groups performed the quantification analysis. Five slides from each brain sample for each antibody and each slide containing eight fields of view from striatum and corpus callosum (CC) were digitized under a 40× objective (Olympus BX40; Olympus America, Center Valley, PA, USA) using a 3-CCD color video camera (Sony DXC-970MD; Tokyo, Japan) interfaced with an MCID image analysis system (Imaging Research, St. Catharines, ON, Canada). Using an MCID image analysis system (Imaging Research, St. Catharines, ON, Canada), numbers of positive cells or positive stained areas were measured using a built-in densitometry function with a density threshold above unstained area set uniformly for all groups.

### Polymerase Chain Reaction (PCR)

For testing the expression of tPA with increasing age, total brain RNA of young, adult and middle-aged WT mice was isolated and utilized to perform quantitative PCR following standard protocol [23]. The samples were tested by an investigator blinded to experimental groups. The following mouse tPA primer was used: FWD: CTGAGGTCACAGTCCAAGCA; REV: ACAGATGC TGTGAGGTGCAG. Relative gene expression was analyzed by the 2^ -ΔΔCt^ method.

**Figure 3.**
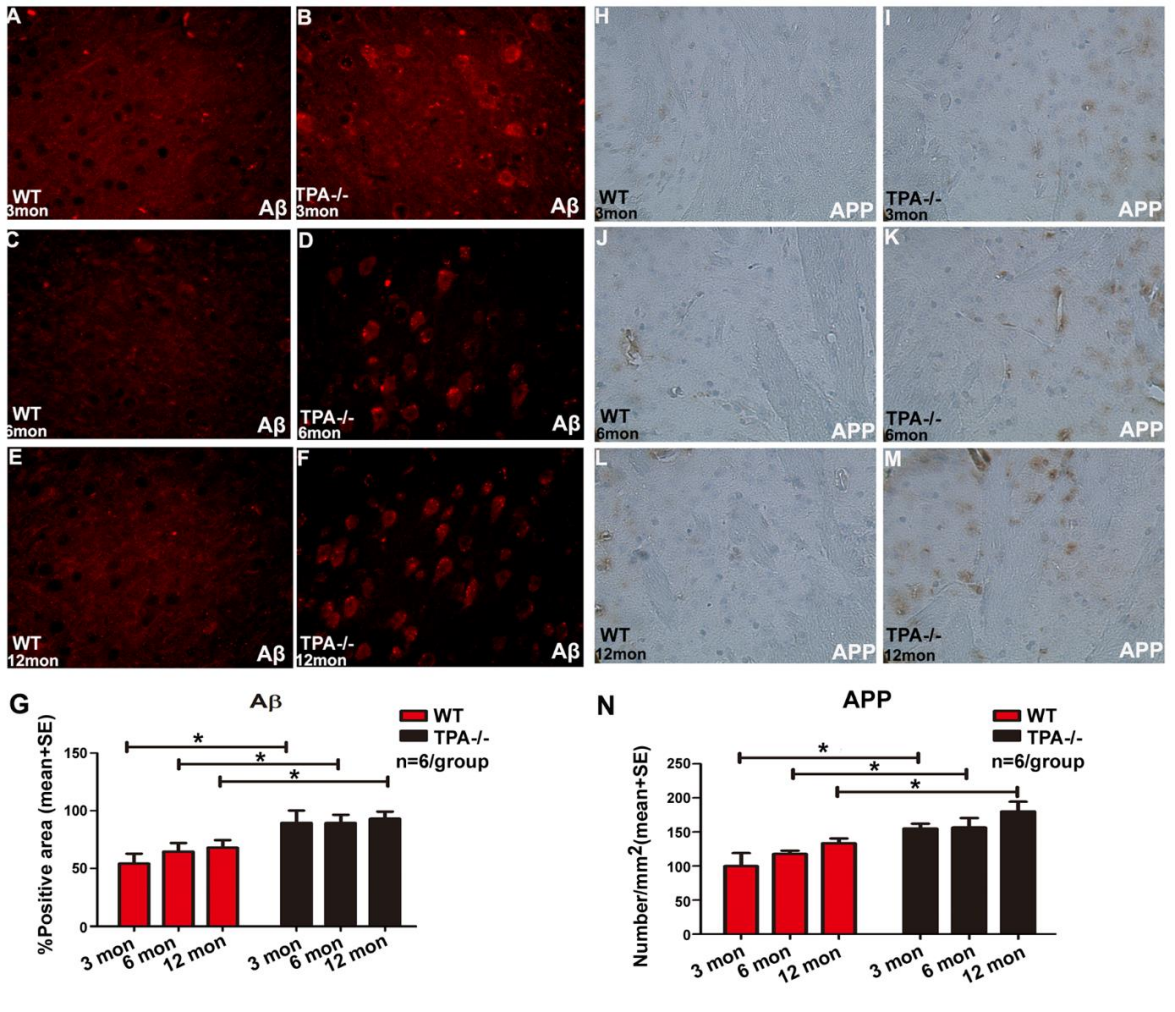
Deficiency of tPA increases age induced Aβ and APP aggregation in the brain (**A-G**) Aβ and (H-N) APP deposition significantly increases in brain of young, adult and middle-aged tPA^-/-^ mice compared to corresponding age matched WT mice. However, no significant age dependent changes were observed within WT or tPA^-/-^ groups of varying age. Aβ and APP quantification data is presented in panels G and N, respectively. *p<0.05, n=6/group.

**Figure 4.**
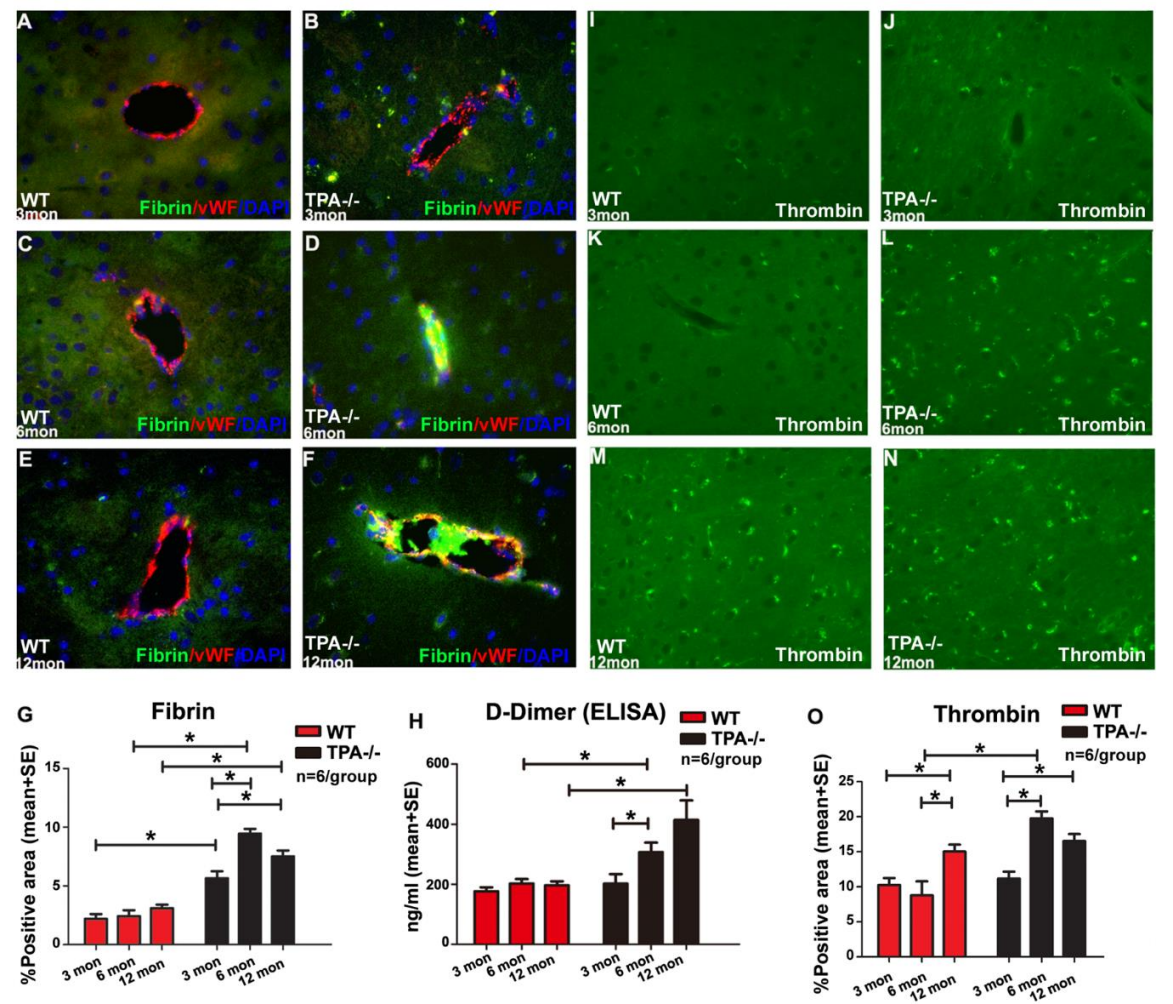
Aging and deficiency of tPA induce increased fibrin deposition in vessels and thrombin deposition in the brain (**A-G**) Fibrin deposition in brain of adult and middle-aged tPA^-/-^ mice significantly increases compared to young tPA^-/-^ mice, and the fibrin accumulation is significantly greater in young, adult and middle-aged tPA^-/-^ mice compared to corresponding age-matched WT mice. Blood vessels are stained using vWF (Von Willebrand factor) immunostaining and DAPI is used to stain nuclei. Quantification data for fibrin immunostaining (A-F) is presented in panel (G. H) ELISA data indicate that D-Dimer expression levels were significantly increased in adult and middle-aged tPA^-/-^ mice compared to age-matched WT mice. (I-O) Thrombin deposition significantly increased in middle-aged WT and tPA^-/-^ mice compared to young and adult WT or tPA^-/-^ mice, respectively. In addition, thrombin deposition was significantly higher in adult tPA^-/-^ mice compared to adult WT mice. Quantification data for thrombin immunostaining (I-N) is presented in panel O. *p<0.05, n=6/group.

### ELISA

To measure thromboembolism, ELISA (From LifeSpan BioSciences, Mouse Fibrin Degradation Product D-Dimer ELISA Kit (Competitive EIA), cat# LS-F6179) was performed to test the level of D-Dimer in serum of young, adult and middle-aged WT and tPA^-/-^ mice using previously described methods [24].

### Glymphatic system measurement

Glymphatic system measurements were performed using previously described methods [16, 25, 26]. Male, adult (6m) tPA^-/-^ and WT mice were employed. 5µl of 1% (diluted in artificial cerebrospinal fluid (CSF)) Texas Red conjugated dextran (MW: 3 kD, Invitrogen) and 5µl of 1% FITC conjugated dextran (MW: 500 kD, Invitrogen) was injected into the cisterna magna over 5 minutes using a syringe pump at a flow rate of 2µl/min. Mice were sacrificed at 30 minutes, 1 hour and 3 hours from the start of infusion and transcardially perfused with saline and 4% formaldehyde (n=6/group/time point). Coronal brain sections were cut using a vibratome (80µm thick) and imaged under a laser scanning confocal microscope. Then, an investigator blinded to the experimental groups employed Image J (NIH) to quantify the fluorescence density (Texas Red and FITC). The fluorescence area of brain was outlined and the % of positive fluorescence area was calculated.

### Golgi staining

Golgi staining was utilized to test the neurite branching and neurite spine density in adult (6m) WT and tPA^-/-^ mice (n=6/group). The Rapid Golgi stain kit (FD Neuro-Technologies, Columbia, MD, USA) was employed following the manufacturer’s protocol. The primary branching of ten intact neurons from each mouse in the layer III of cortex were counted under a 40× objective. Ten intact neurons from each mouse were chosen in layer III of cortex and CA3 region of hippocampus and the neurite spine density was counted under an oil immersion 100× objective on 10 secondary dendrites of at least 10 mm length [17]. All measurements were performed by an investigator blinded to the experimental groups.

### Statistical Analysis

One-way Analysis of Variance (ANOVA) was utilized for evaluation of cognitive functional outcome and histology. If an overall treatment group effect was detected at p<0.05, pair-wise comparisons were made. All data are presented as mean ± standard error (SE).

**Figure 5.**
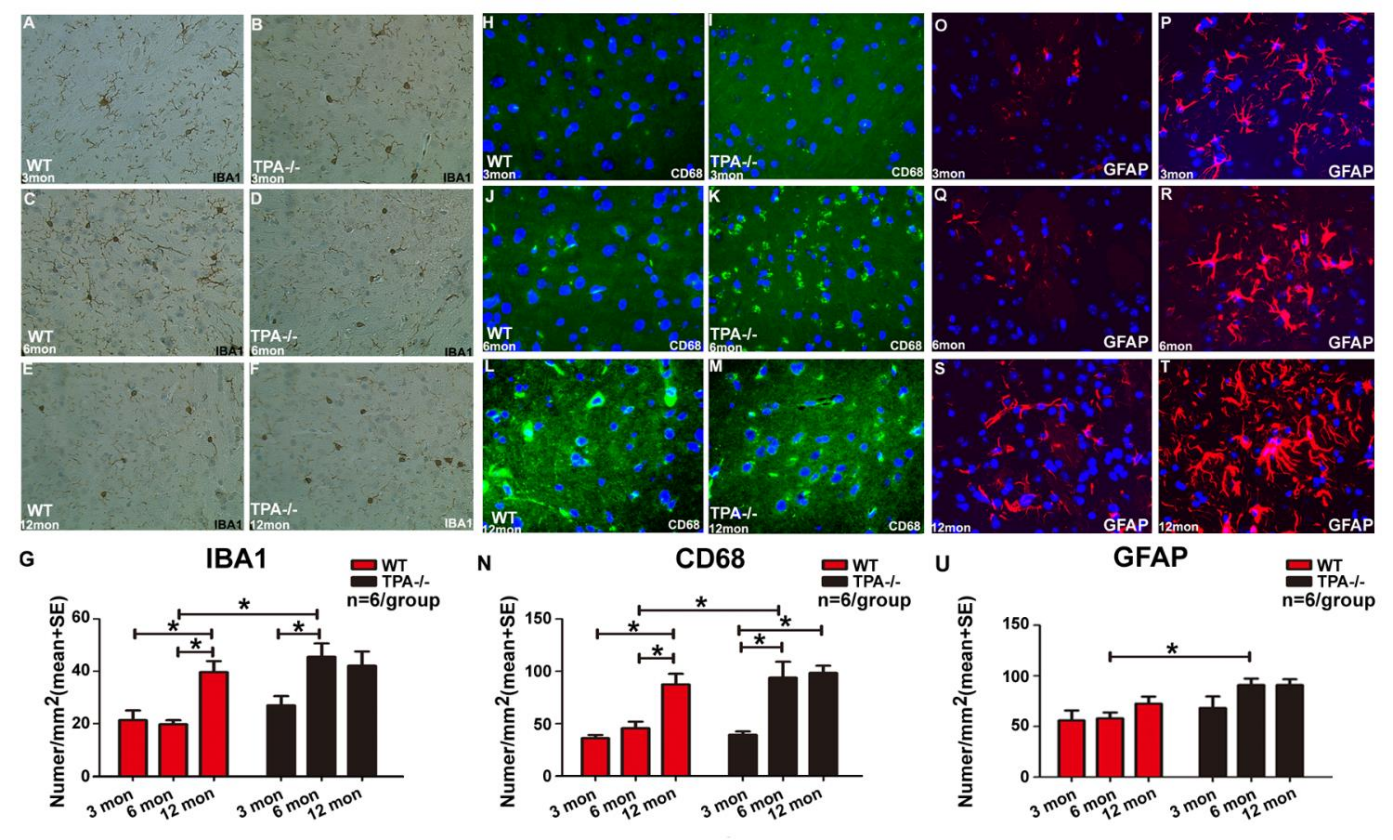
Aging and deficiency of tPA increases number of microglia, astrocytes and macrophages Immunostaining and quantification data indicate that middle-aged WT and tPA^-/-^ mice have significantly increased (**A-G**) number of microglia (IBA1) and (**H-N**) macrophages (CD68) compared to young WT or tPA^-/-^ mice, respectively. In addition, adult tPA^-/-^ mice exhibit significantly increased numbers of microglia (IBA1, **A-G**), macrophages (CD68, **H-N**) and astrocytes (GFAP, **O-U**) compared to adult WT mice. Quantification data for microglia, macrophages and astrocytes are presented in panels G, N and U respectively. *p<0.05, n=6/group.

## RESULTS

### Aging decreases brain tPA expression and induces cognitive impairment

To test whether aging evokes reduced cognitive outcome, odor test, novel objective recognition test and Morris water maze tests were performed in young, adult and middle-aged WT mice. Figure 1A-B shows that middle-aged WT mice have significant short-term memory deficits indicated by lower discrimination index in the novel object recognition and odor tests indicating poor short term and overnight memory compared to young WT mice. To test whether age affects tPA expression in the brain, PCR was employed. Figure 1C shows that adult mice have lower tPA expression in brain compared to young mice, and middle-aged mice have significantly lower tPA levels than young and adult mice. Our data indicate that there is an age-dependent decline in cognitive function and tPA expression levels in brain in WT mice.

**Figure 6.**
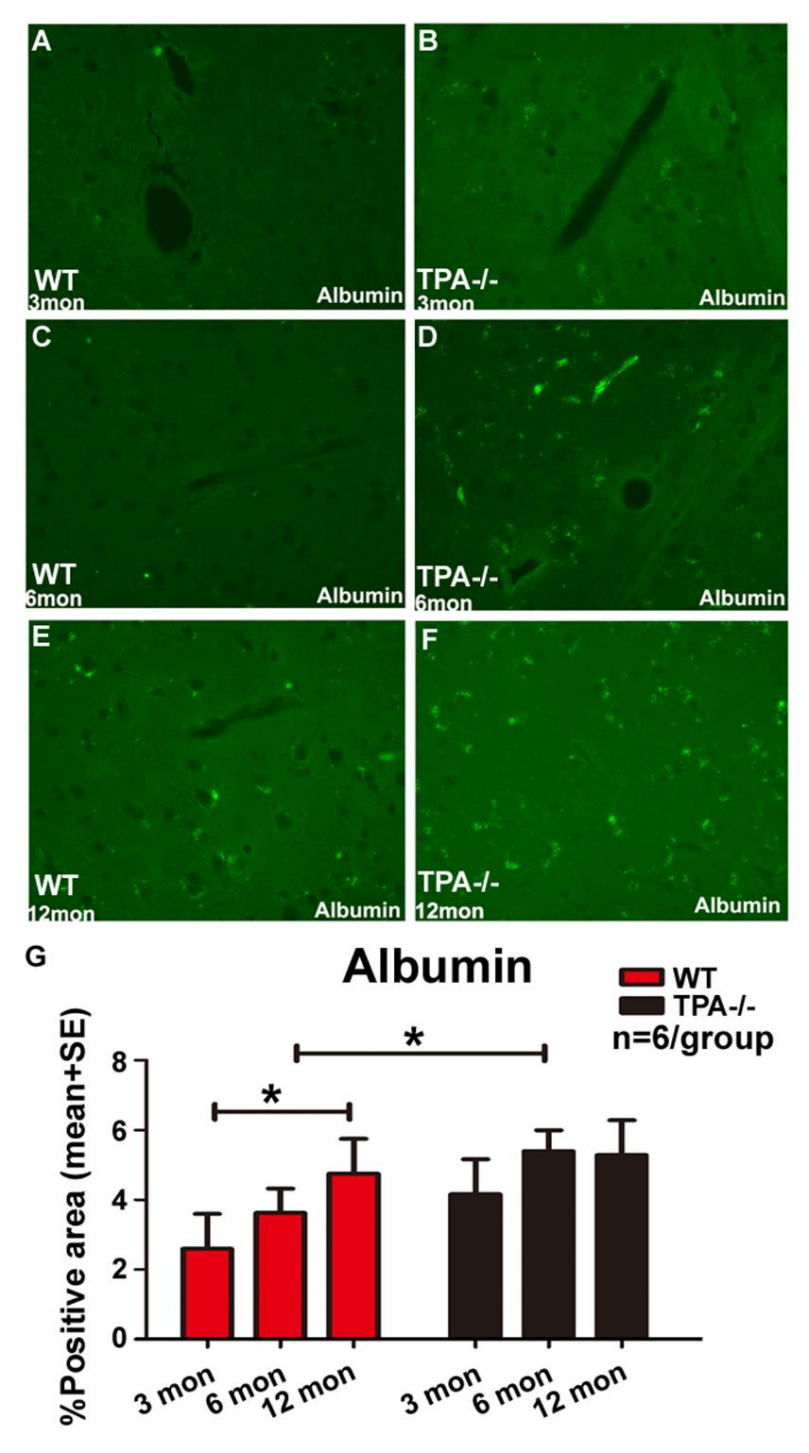
Aging and deficiency of tPA induces BBB disruption BBB leakage (Albumin) is significantly increased in middle-aged WT mice compared to young WT mice and adult tPA^-/-^ mice compared to adult WT mice. Quantification data for albumin immunostaining (**A-F**) is presented in panel G. *p<0.05, n=6/group.

To test whether tPA mediates the age-dependent cognitive decline, young, adult and middle-aged tPA^-/-^ mice were subjected to cognitive evaluation. Compared to age-matched WT mice, middle-aged tPA^-/-^ mice show increased significant short-term memory loss (novel object recognition test), and young, adult and middle-aged mice exhibit significant long term (odor test) memory deficits (Fig. 1A-B). In the Morris water maze test (Fig. 1D), compared to age-matched WT mice, young, adult and middle-aged tPA^-/-^ mice exhibit significant spatial learning and memory deficits indicated by significantly increased latency to reach submerged platform, significantly reduced time spent in platform quadrant, and increased path length, without differences in swim speed i.e. motor function.

### Aging and tPA deficiency increase WM damage and increase APP and Aβ deposition

To investigate whether tPA deficiency alters WM integrity, axon and myelin densities were measured in the brain WM. Figure 2 shows that axon and myelin densities are significantly decreased in middle-aged WT and tPA^-/-^ mice compared to young and adult WT and tPA^-/-^ mice, respectively. Additionally, young, adult and middle-aged tPA^-/-^mice exhibit increased axon loss but not myelin loss, compared with age-matched WT mice. Since onset of WM damage in tPA^-/-^ mice is evident at 6m of age compared to WT mice, Golgi staining was performed in adult (6m) WT and tPA^-/-^ mice. Figure 2O-T indicate that there is a significant decrease in spine density in adult tPA^-/-^ mice compared to adult WT mice, but the primary neuronal branching count did not change. To further explore mechanism of tPA-induced cognitive decline, deposition of Aβ and APP in the brain were measured. Figure 3 shows that tPA deficiency but not aging, aggravates Aβ and APP deposition in the brain. Compared with age-matched WT mice, young, adult and middle-aged tPA^-/-^ mice exhibit significantly increased APP expression and Aβ deposition in brain.

**Figure 7.**
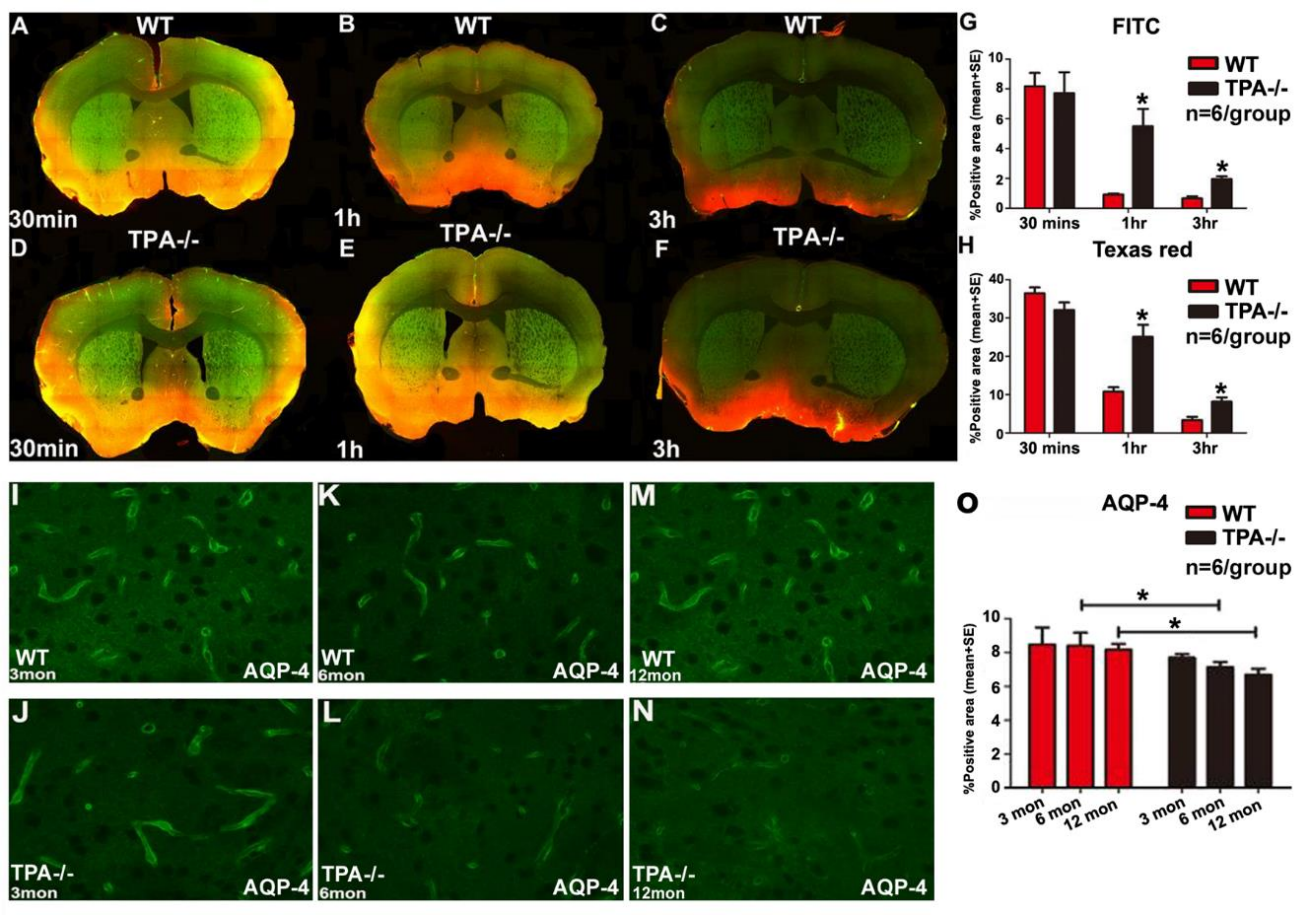
Deficiency of tPA decreased AQP-4 expression and induced glymphatic system dysfunction (**A-H**) Compared with adult WT mice, adult tPA^-/-^ mice exhibit significantly delayed clearance of dyes injected into the cisterna magna indicating impaired functioning of the glymphatic system. (**I-O**) AQP-4 expression was significantly decreased in adult and middle-aged tPA^-/-^ mice compared to age matched WT mice respectively. Quantification data for AQP-4 immunostaining is presented in panel O. *p<0.05, n=6/group.

### Aging and tPA deficiency increase fibrin and thrombin deposition in the brain

Figure 4A-G shows that while there are no differences in fibrin deposition in WT mice with increasing age. Adult and middle-aged tPA^-/-^ mice have significantly increased perivascular fibrin deposition compared to young tPA^-/-^ mice as well as compared to corresponding age-matched WT control mice. Figure 4I-O shows that middle-aged WT and tPA^-/-^ mice have increased thrombin deposition compared to young and adult WT and tPA^-/-^ mice, respectively. In addition, compared to adult WT mice, adult tPA^-/-^ mice exhibit significantly increased thrombin deposition. As tPA plays an important role in regulating thrombus formation, we investigated occurrence of thromboembolism by measuring D-Dimer expression in serum and found that D-Dimer was significantly increased in adult tPA^-/-^ mice compared to young tPA^-/-^ mice. In addition, adult and middle-aged tPA^-/-^ mice exhibit significantly increased D-Dimer expression compared to age-matched WT mice (Fig. 4H).

### Aging and tPA deficiency increase BBB permeability and neuroinflammation

To investigate whether aging and tPA deficiency aggravate neuroinflammation in the brain, macrophage (CD68), microglia (IBA1) and astrocyte (GFAP) expression was quantified. Figure 5 shows a significant increase in the levels of microglia and macrophage expression in adult and middle-aged WT and tPA^-/-^ mice as compared with young WT and tPA^-/-^ mice, respectively. In addition, the numbers of microglia, astrocytes and macrophages were elevated in adult tPA^-/-^ mice compared to adult WT mice. To test whether aging and tPA deficiency affects BBB integrity, FITC-albumin immunostaining was performed. Figure 6 shows that there is a significant increase in BBB leakage in middle-aged WT mice compared to young WT mice. BBB disruption is also significantly increased in adult tPA^-/-^ mice compared to adult WT mice.

### tPA deficiency decreases AQP-4 expression and induces glymphatic dysfunction in adult mice

To test whether impairment of waste clearance pathways such as the glymphatic system may contribute towards aging and tPA deficiency induced neuroinflammation and accumulation of neurotoxins in the brain, we employed ex-vivo fluorescence microscopy to study glymphatic waste clearance pathways. Since the water channel protein AQP-4 facilitates glymphatic clearance pathways, we measured AQP-4 expression around cerebral blood vessels in WT and tPA^-/-^ mice. Figure 7 shows that adult and middle-aged tPA^-/-^ mice exhibit significantly reduced perivascular AQP-4 expression compared to age-matched WT mice. Therefore, adult WT and tPA^-/-^ mice were employed to test glymphatic function. Figure 7 shows that adult tPA^-/-^ mice exhibit significantly delayed waste clearance compared to adult WT mice indicated by greater dye retention at 1hr and 3hr time points.

### DISCUSSION

In this study, we found that aging induces progressive cognitive impairment and decreases tPA expression in brain. tPA deficiency in mice induces significant cognitive impairment, axonal damage, and increased deposition of neurotoxins in the brain. tPA deficiency in adult mice significantly increases BBB permeability, neuroinflammation, and impairs glymphatic system function compared to adult WT mice. The increase in deposition of neurotoxins in the brain, WM damage, and cognitive impairment associated with tPA deficiency in aging mice, may in part, be attributed to diminished perivascular AQP-4 and impaired glymphatic waste clearance pathways.

In the aging population, there are several forms of neurodegenerative disorders, including vascular dementia and Alzheimer’s disease [27]. Decreased tPA activity is associated with a concomitant reduction in clearance of Aβ which plays an important role in the pathogenesis of Alzheimer’s disease [28]. Results of a post mortem study indicate that the mean Aβ level is twice as high in vascular dementia than age-matched controls and twice as high in Alzheimer’s disease compared to vascular dementia [29]. In APP transgenic mice, increased Aβ in the brain has been correlated to elevated levels of neuroserpin which is an effective inhibitor of tPA. Neuroserpin, a serine protease inhibitor, is increased in Alzheimer’s disease [30]. Knockout of neuroserpin in mice decreases Aβ levels in the brain [31]. Previous studies have shown that brain tPA expression is down regulated with aging which reinforces Alzheimer’s disease [32, 33]. tPA has been also reported to mediate learning, anxiety-like behavior and drug dependency [34]. tPA participates in neuronal migration, synaptic plasticity and neurite outgrowth [34], therefore, tPA deficiency likely contributes to cognitive impairment. WM damage is known to induce cognitive impairment [35, 36]. Administration of tPA improves axonal remodeling and functional recovery after stroke [37]. In this study, we found that aging decreases brain tPA levels and induces progressive cognitive impairment. tPA^-/-^ mice exhibit significantly increased cognitive deficits, and axonal/WM damage which increases with age, when compared to WT mice. Therefore, there may be a potential link between aging, tPA deficiency, axonal damage and cognitive decline which remains relatively unexplored and could present a novel therapeutic target to treat aging related cognitive decline.

Overexpression of Aβ derived from APP is a hallmark of Alzheimer's disease and is associated with cognitive decline and neuroinflammation [38]. APP is produced in bulk in neurons and is rapidly metabolized [39]. APP proteolysis involves multiple alternative pathways, and APP not only generates Aβ and soluble APP, but also acts as a marker of fast axonal transport [40-43]. APP accumulation in the brain has been extensively evaluated as a sensitive marker for the detection of axonal injury [40-43]. Deposition of fibrin not only contributes to neurodegeneration and neuroinflammation, but may also mimic the vascular pathology of Alzheimer’s disease [9, 44]. Thrombin has an essential role in the blood coagulation cascade, and contributes to BBB dysfunction and neuroinflammation which are early features of Alzheimer's disease [45]. Accumulation of Aβ may be increased by thrombin through cleavage of APP, increasing oxidative stress and intracellular Ca^2+^ influx [46]. tPA^-/-^ mice subject to stroke exhibit increased fibrin deposition, cerebral blood flow decline, distensible infarct size and greater neurological deficits, suggesting that tPA deficiency may promote fibrin deposition in the vessels and induce BBB degeneration and neuroinflammation [47]. Inflammation and immune response after stroke play an essential role in determining stroke outcome [48]. tPA also increases proinflammatory macrophage activation [49] and survival and recruitment of microglia [50, 51]. AD pathology involves a variety of risk factors that provoke degeneration of vessels and BBB [52, 53], which could worsen cognitive deficits [54]. D-Dimer is a fibrin degradation product, and high D-Dimer expression levels in serum imply the presence of high levels of fibrin degradation products indicating thromboembolism and subsequent fibrinolysis. In a study including elderly participants (>65 years old), the risk of VaD was found to increase with increasing plasma D-Dimer levels [11]. Another study identified elevated plasma D-Dimer and other coagulation and inflammatory serum markers in VaD patients [55]. Hemostasis abnormalities such as increased plasma plasminogen activator inhibitor type 1 (PAI-1), D-Dimer, fibrinogen, and von Willebrand factor (vWF, a marker of endothelial disturbance) were more frequently and significantly observed in patients with VaD than with AD [56, 57]. In the present study, our data indicate that adult tPA^-/-^ mice have significantly greater BBB disruption, and significantly increased numbers of microglia and macrophages in the brain. In addition, young, adult as well as middle-aged tPA^-/-^ mice have increased deposition of toxic substances in the brain such as Aβ, APP, fibrin, D-Dimer and thrombin (adult only) compared to age-matched WT mice. However, whether tPA directly influences the activation or migration of microglia and macrophage is not clear. Increased inflammatory cell infiltration may be due to disruption of BBB and deposition of neurotoxins in the brain. Deposition of neurotoxins may also induce WM damage, neuroinflammation and cognitive impairment.

**Figure 8.**
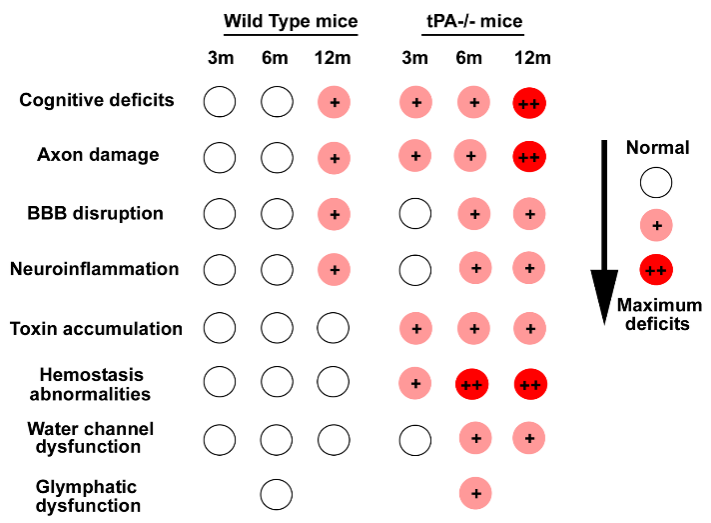
Summary scheme indicating age-dependent changes in wild type and tPA^-/-^ mice.

Glymphatic dysfunction has been reported in several diseases and may lead to a variety of neurodegenerative, neurovascular and neuroinflammatory changes in the brain [17, 58-60]. AQP-4 which is mainly expressed on astrocytic end feet plays an essential role in glymphatic system function [61]. Increased deposition of Aβ and larger amyloid plaque were associated with decreased tPA level or proteolytic activity [2, 62]. Our data indicate that tPA deficiency in adult mice significantly impairs function of the glymphatic system and there is decreased AQP-4 expression and delayed waste clearance compared to adult WT mice. Thus, deposition of Aβ, thrombin, fibrin and APP may at least in part be attributed to the dysfunction of glymphatic system, and increased thrombin has the ability to further deteriorate glymphatic system by inhibiting AQP-4 expression [63]. Compared to WT mice, young tPA^-/-^ mice did not exhibit AQP-4 loss, hemostasis abnormalities (D-dimer, thrombin) or severe neuroinflammation (IBA1, CD68 or GFAP) which were evident in adult and middle-aged mice. Therefore, we tested function of the glymphatic system in adult WT and tPA^-/-^ mice. However, we cannot exclude the possibility of impaired glymphatic system function in young mice, and further studies are warranted.

Our data indicate that tPA deficiency may contribute to aging induced WM damage, neuroinflammation, glymphatic dysfunction, accumulation of toxins in the brain which in concert, lead to cognitive deficits as summarized in Figure 8. We have not investigated which pathological mechanisms dominate aging induced tPA deficiency mediated cognitive dysfunction. The interaction among inflammatory responses, BBB leakage and glymphatic system dysfunction warrant further investigation. Since dementias are prevalent in elderly, future studies with aged (>18 months) male and female mice are warranted.

### Conclusions

tPA may play an important role in contributing to aging induced cognitive decline, axonal/WM damage, BBB disruption and glymphatic dysfunction in the brain.
